# Macrostructural brain alterations at midlife are connected to cardiovascular and not inherited risk of future dementia: the PREVENT-Dementia study

**DOI:** 10.1007/s00415-022-11061-7

**Published:** 2022-03-13

**Authors:** Maria-Eleni Dounavi, Coco Newton, Natalie Jenkins, Elijah Mak, Audrey Low, Graciela Muniz-Terrera, Guy B. Williams, Brian Lawlor, Lorina Naci, Paresh Malhotra, Clare E. Mackay, Ivan Koychev, Karen Ritchie, Craig W. Ritchie, Li Su, John T. O’Brien

**Affiliations:** 1 Department of Psychiatry, School of Clinical Medicine, University of Cambridge, Box 189, Level E4 Cambridge Biomedical Campus, Cambridge, CB2 0SP UK; 2 Centre for Dementia Prevention, University of Edinburgh, Edinburgh, UK; 3 Department of Clinical Neurosciences and Wolfson Brain Imaging Centre, University of Cambridge, Cambridge, UK; 4 Institute of Neuroscience, Trinity College Dublin, University of Dublin, Dublin, Ireland; 5 Division of Brain Science, Imperial College Healthcare NHS Trust, London, UK; 6 Department of Psychiatry, Oxford University, Oxford, UK; 7 INM, Univ Montpellier, INSERM, Montpellier, France; 8 Department of Neuroscience, University of Sheffield, Sheffield, UK

**Keywords:** Alzheimer’s disease, Cortical thickness, APOE, Hippocampal subfields, Dementia risk

## Abstract

**Background:**

Macrostructural brain alterations in the form of brain atrophy or cortical thinning typically occur during the prodromal Alzheimer’s disease stage. Mixed findings largely dependent on the age of the examined cohorts have been reported during the preclinical, asymptomatic disease stage. In the present study, our aim was to examine the association of midlife dementia risk with brain macrostructural alterations.

**Methods:**

Structural 3T MRI scans were acquired for 647 cognitively normal middle-aged (40–59 years old) participants in the PREVENT-Dementia study. Cortical thickness, volumes of subcortical structures, the hippocampus and hippocampal subfields were quantified using Freesurfer version 7.1. The clarity of the hippocampal molecular layer was evaluated based on T2-weighted hippocampal scans. Associations of structural measures with apolipoprotein ε4 (APOE4) genotype and dementia family history (FHD), were investigated using linear regression. Correlations between the CAIDE dementia risk score (incorporating information about blood pressure, cholesterol, physical activity, body mass index, education, age and sex) and structural measures were further investigated.

**Results:**

A higher CAIDE score was associated with thinner cortex and a larger hippocampal fissure. APOE4 genotype was associated with reduced molecular layer clarity.

**Conclusions:**

Our findings suggest that a higher CAIDE score is associated with widespread cortical thinning. Conversely, APOE4 carriers and participants with FHD do not demonstrate prominent macrostructural alterations at this age range. These findings indicate that cardiovascular and not inherited risk factors for dementia are associated with macrostructural brain alterations at midlife.

## Introduction

Neurodegeneration, one of the main pathological hallmarks of Alzheimer’s disease (AD), can be evaluated with structural MRI and involves a characteristic pattern of gray matter (GM) atrophy in key temporo-parietal regions [1]. A more detailed investigation of the atrophic pattern has proposed four distinct sub-types: typical AD (hippocampal and cortical atrophy); hippocampal sparing; limbic predominant and no atrophy, with these subtypes demonstrating different clinical progression rates [2, 3].

The majority of studies investigating neurodegeneration with structural MRI have focused on established AD, mild cognitive impairment (MCI) or subjective cognitive decline, with very few studies conducted in the disease’s preclinical stage where participants are cognitively asymptomatic, and nearly all of these have been in older people (65 years or over) [4]. These later studies on preclinical AD, especially when focused on young or middle-aged participants, do not normally include information on disease progression, since this would take many years, even decades. Hence, they utilize established risk stratification approaches, using factors such as family history of dementia (FHD) or apolipoprotein (APOE) ε4 genotype, which is the main genetic risk factor for sporadic late onset AD—LOAD [5]. Lifestyle risk factors are also used to determine individuals at risk of future dementia. There is growing evidence to suggest that up to 40% of all dementia cases are associated with known modifiable risk factors [6]. Several dementia risk scores incorporating lifestyle risk factors have been devised [7–9]. Amongst them, the Cardiovascular Risk factors aging and dementia (CAIDE) score has been optimized for middle-aged populations [9] and has been validated in a large US population followed longitudinally over 40 years [10].

Findings on structural alterations during the preclinical AD phase vary, ranging from absence of alterations to subtle volumetric differences or even unexpected patterns of hyper-trophy, with the results heavily depending on age [4]. In studies of middle-aged participants, mixed observations have been reported. In a middle-aged cohort (mean age 58), it was found that the APOE4 genotype was associated with both lower hippocampus and higher thalamus volumes [11]. Further structural analysis revealed atrophy in the signature-AD region and differential associations between cognition and local brain volume as mediated by APOE4 genotype [12]. In a cohort of similar age, subjects with APOE4, FHD or both demonstrated subtle atrophy patterns [13]. Individuals with a maternal AD family history in late midlife, demonstrated widespread patterns of brain atrophy [14]. In midlife subjects unaware of cognitive decline higher regional GM volumes have been reported [15]. Cross-sectional studies investigating hippocampal subfield volumes in middle-aged APOE4 carriers have reported no differences or subtle patterns of atrophy in the cornu ammonis 3/dentate gyrus (CA3/DG) and stratum radiatum/stratum lacunosum-moleculare (SRLM) [16–18].

In addition to investigating volumetric alterations, structural MRI indices such as cortical thickness, have shown promise in detecting anatomical alterations in established AD, prodromal AD and preclinical disease stages [19, 20]. In early stages or in carriers of the APOE4 gene, both higher [21, 22] and lower thickness [23, 24] as well as no differences [25, 26] have been reported. Longitudinal studies investigating rates of change on cortical thickness have reported an accelerated cortical thinning in APOE4 carriers [21, 27].

Establishing the neurodegenerative profile in middle-aged participants at risk of future dementia is of paramount importance for planning of interventional studies and clinical trials since it allows to evaluate whether macrostructural alterations have already occurred. Our aim in the present study was to investigate alterations in cortical thickness and patterns of localized atrophy in the middle-aged PREVENT-Dementia cohort using three different risk stratification approaches: APOE4 genotype, FHD and CAIDE score. Our hypothesis was that we would observe cortical thinning in areas known to be influenced in early stages of the disease such as the entorhinal and perirhinal cortices in high-risk participants. In terms of volumetrics, we focused our analysis on subcortical structures and the hippocampus and hypothesized no group differences. For the hippocampal subfield analysis, our hypothesis was that APOE4 carriers would have a lower molecular layer volume based on previous observations in the cohort [28]. We further hypothesized that the molecular layer would be less clearly delineated in high-risk groups. Finally, we hypothesized that we would observe significant age x risk factor (APOE4, FHD) interactions in predicting regional volumes and thickness.

## Methods

### Cohort

701 participants were recruited in the PREVENT-Dementia study from five study sites: West London, Edinburgh, Cambridge, Oxford and Dublin. The main entry criteria were age between 40 and 59 and absence of dementia or other neurological disorders. The primary risk stratification approach was dementia family history defined by having one or both parents with dementia (50–50 recruitment target for those with and without FHD). Participant APOE genotype analysis was carried out on QuantStudio12K Flex to establish APOE variants. APOE information was not collected for five participants. The CAIDE score was calculated based on published thresholds on age, education, blood pressure, activity, cholesterol, body mass index and did not incorporate APOE4 genotype; in particular, we have used model 1 from Kivipelto et al. [9]. Overall, 23 participants had missing information for CAIDE calculation.

**Fig. 1:**
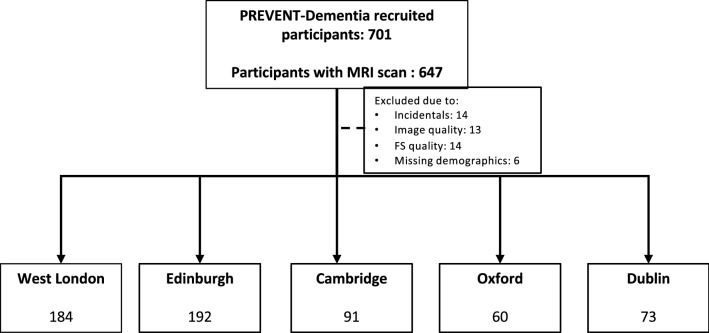
Structural scans used in the present analysis per PREVENT-Dementia site. Scans were excluded either due to incidental findings or due to poor scan quality that could potentially impact the structural analyses pipelines. Further exclusions were applied due to quality of the Freesurfer (FS) outcome or missing demographics

A detailed description of all the data acquired as part of the study can be found in [29, 30]. From the 701 recruited participants, 647 had an MRI scanning session at baseline (Fig. 1). 27 scans were excluded from analysis after visual inspection either due to poor quality, artifacts (e.g. excessive motion, poor contrast) or incidental findings (e.g. meningiomas). An estimated years to dementia onset (EYO) variable was calculated for participants with dementia family history, based on the difference between the age of the participant and the age of parental dementia diagnosis (if both parents had dementia, the younger onset was used) and had a mean of 22.8 years.

### MRI protocol

As part of the PREVENT-Dementia MRI protocol, a T1-weighted magnetization prepared rapid gradient echo (MPRAGE) (repetition time = 2.3 s, echo time = 2.98 ms, 160 slices, flip angle = 9°, voxel size = 1 mm^3^ isotropic) and a T2-weighted hippocampal (repetition time = 6.42 s, echo time = 11 ms, 20 slices, flip angle = 160°, voxel size = 0.4 × 0.4 × 2.0 mm) scan were acquired. All study sites used 3T Siemens scanners and in particular, the following models: Prisma (Oxford, Edinburgh), Prisma fit (Cambridge), Verio (West London, Edinburgh) and Skyra (Dublin; Edinburgh). All scans were corrected for field inhomogeneities using the Advanced Normalisation Toolbox (ANTs) N4 algorithm [31].

### Surface-based analysis

Freesurfer version 7.1.0 was used for data processing [32]. The *recon-all* pipeline was run for every subject with standard settings. The brain masks and surfaces were inspected following recon-all and manual corrections were applied: (a) in the form of erosion of non-brain voxels from the brain mask or non-WM voxels from the WM mask, (b) in the form of filling of areas where the brain was not correctly identified or (c) with the addition of control points in cases where white matter was not successfully identified. Manual corrections were applied for the majority of subjects (87%). We quantified cortical thickness in a vertex wise level and in 68 regions based on the Desikan-Killiany atlas [33]. Furthermore, the volume of bilateral hippocampi, thalami, amygdala, putamen, caudate, accumbens and pallidum were quantified. Values from the left and the right hemisphere were averaged for cortical thickness and added up for volumetric analysis.

### Hippocampal subfield segmentation

A dedicated Freesurfer hippocampal subfields segmentation module was applied using information from both the T1 and T2-weighted images. Hippocampal subfield segmentation relies on an atlas constructed based on in-vivo and high-resolution post-mortem scans and on manual subfield delineation from experienced radiographers [34]. In particular, the Freesurfer algorithm utilizes the constructed probabilistic atlas and the individual voxel intensities to proceed to subfield segmentation using Bayesian inference [34].

Following previous methodology, we concatenated subfields in the following: hippocampal fissure, hippocampal tail, subiculum (subiculum + presubiculum + parasubiculum), CA4/DG (CA4 + GC_ML_DG), CA1, molecular layer (ML) and CA3 [28]. We have also combined the left and the right hemispheres.

**Fig. 2:**
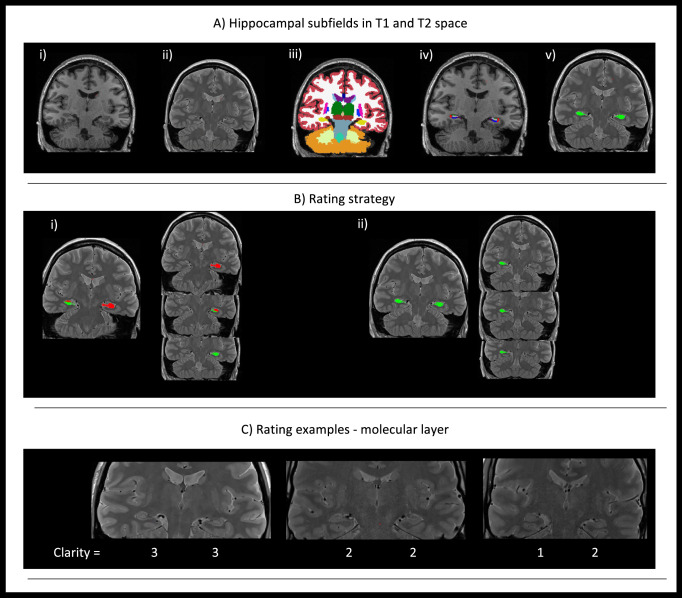
Pipeline for molecular layer clarity rating. **A** The T1 (i) and T2-weighted (ii) images are shown for one subject. In (iii) the outcome of the recon-all Freesurfer pipeline is shown followed by an overlay of the generated hippocampal subfields based on both T1 and T2-weighted scans (iv). In (v) the head-body-tail segmented hippocampus has been registered to the T2 space and is used as a guide for the subsequent molecular layer rating. In **B** the three rated slices per hemisphere are shown based on the slices starting from the one where the hippocampal body is the majority of the slice. For this particular subject all slices have been rated with a ‘3’ in a scale of 1–3. In **C** rating examples are shown. A three is assigned when the molecular layer is clearly seen in 80% of the slice and the contrast is satisfactory. A two is assigned when less than 80% is seen or if the contrast is not satisfactory and the molecular layer not clearly seen. A one is assigned when most of the molecular layer is not seen and the contrast is too poor

The molecular layer is a structure of particular interest since it covers the CA1-SRLM area, a region rich in synapses from CA3 and the entorhinal cortex to CA1 [35]. CA1-SRLM is known to be susceptible to tau accumulation in early disease stages [17, 36]. In typically acquired T1-weighted image, it is not discernible, however, when tailored T2-weighted acquisitions are applied, it appears as a dark band due to its myelin content [37]. It has been shown that its clarity reduces in Alzheimer’s disease [38], hence we proceeded in the evaluation of its clarity in the cohort. Toward that end, the T2w images were quality checked and a visual rating on the clarity of the molecular layer was recorded ranging from 1 to 3. The dataset was split in three and was rated by three independent raters (MED, CN, NJ). Agreement was evaluated using Cohen’s κ for 60 scans. For each hemisphere starting from the slice where the body of the hippocampus was dominant and for another two slices posteriorly, we rated clarity per slice and also overall clarity per hemisphere, as follows: 1—non-clear delineation less than 20% can be seen; 2—more than 20% can be seen but not perfectly discernible and 3—clearly seen. The starting slice per hemisphere, was defined by registration of the head-body-tail segmentations generated by the Freesurfer pipeline to the T2 space using the linear transform generated as part of the main pipeline. The pipeline and example ratings are shown in Fig. 2. For this part of the analysis, a further 60 subjects were excluded due to absence of a high-resolution T2 acquisition, poor T2 image quality or due to issues with between-modality registration.

### Statistical methodology

Statistical analysis was conducted in Matlab 2021b (R2021b, The MathWorks Inc., Natick, MA, USA). Wilcoxon rank sum test and *χ*^2^ test were used for between-group comparisons of demographic factors.

The ComBat harmonization algorithm was used for parametric adjustment of the structural measures to account for inter-site differences [39]. This method has been shown to remove site-differences when preserving the relevance of covariates of interest [39, 40]. For the present implementation, we explicitly included as modulators in ComBat: APOE4, FHD, years of education, age and sex. Data from 6 participants were excluded due to missing information (5 APOE; 1 education).

Linear regression models were used to investigate independently the association of structural measures with FHD and APOE4 genotype. Age, sex and years of education were added as predictors to the models. When volumetric measures were examined, eTIV was also included as a predictor. We additionally investigated interactions of APOE4 and FHD with age. Further sub-analysis was conducted to investigate the impact of carrying one or two copies of the APOE4 gene by creating a three-group variable (no_APOE4, APOE4_1, APOE4_2).

CAIDE score is a discrete variable ranging from 0 to 15 and was non-normally distributed in our cohort. It incorporates all risk factors which would be used normally as analysis covariates (e.g. age, sex, education), hence, to investigate the association of CAIDE with cortical thickness, Spearman correlations were run between the CAIDE and regional thickness. To examine associations with volumetric measures partial Spearman correlations controlling for eTIV were used.

For the volumetric comparisons as well as the cortical thickness analysis within the 34 regions of the Desikan-Killiany atlas, the false discovery rate (FDR) method was used to correct for multiple comparisons [41]. Normality, autocorrelation and homoskedasticity of standardized residuals were checked with the Kolmogorov–Smirnov, Ljung-Box Q-test and Engle test, respectively.

The molecular layer clarity was investigated in relation to age, sex, education, APOE4 genotype and FHD in a single linear regression model.

## Results

Demographic specifications for the whole cohort with analysable MRI data can be found in Table 1. In addition to the excluded datasets due to imaging issues and missing information, a further 14 datasets were excluded due to issues with the automated pipeline. Participants without FHD (FHD–) were slightly younger than participants with FHD (FHD+) and APOE4 carriers (APOE4+) were younger than non-carriers (APOE4–). As would be expected, the FHD+ group had significantly more APOE4 carriers.

**Table 1:** Demographic specifications of the analysable cohort

	Whole cohort (*n* = 600)
Age (years)	51.2 ± 5.4
Sex (% females)	61.3%
Education (years)	16.7 ± 3.4
Systolic blood pressure (mmHg)	125.3 ± 15.7
BMI (kg/m^2^)	27.6 ± 5.4
APOE4 (% carriers)	38.8%
*Homozygotes*	5.3%
CAIDE^a^	4.8 ± 2.5
EYO (years)	22.8 ± 7.3

	FHD− (*n* = 281)	FHD+ (*n* = 319)	*p*-value
Age	50.6 ± 5.9	51.8 ± 4.9	0.03
Sex	58.4%	64.0%	0.16
Years of education	16.9 ± 3.6	16.5 ± 3.1	0.27
APOE4	32.5%	44.6%	< 0.01

	APOE4− (*n* = 367)	APOE4+ (*n* = 233)	*p*-value
Age	51.6 ± 5.4	50.6 ± 5.5	0.02
Sex	61.3%	61.4%	0.98
Years of education	16.6 ± 3.3	16.8 ± 3.4	0.67

Shown values are mean ± standard deviation or percentages

APOE, apolipoprotein e4; EYO, estimated years to dementia onset

^a^Missing information for 23 participants

### Association of risk factors with regional volumes and cortical thickness

**Fig. 3:**
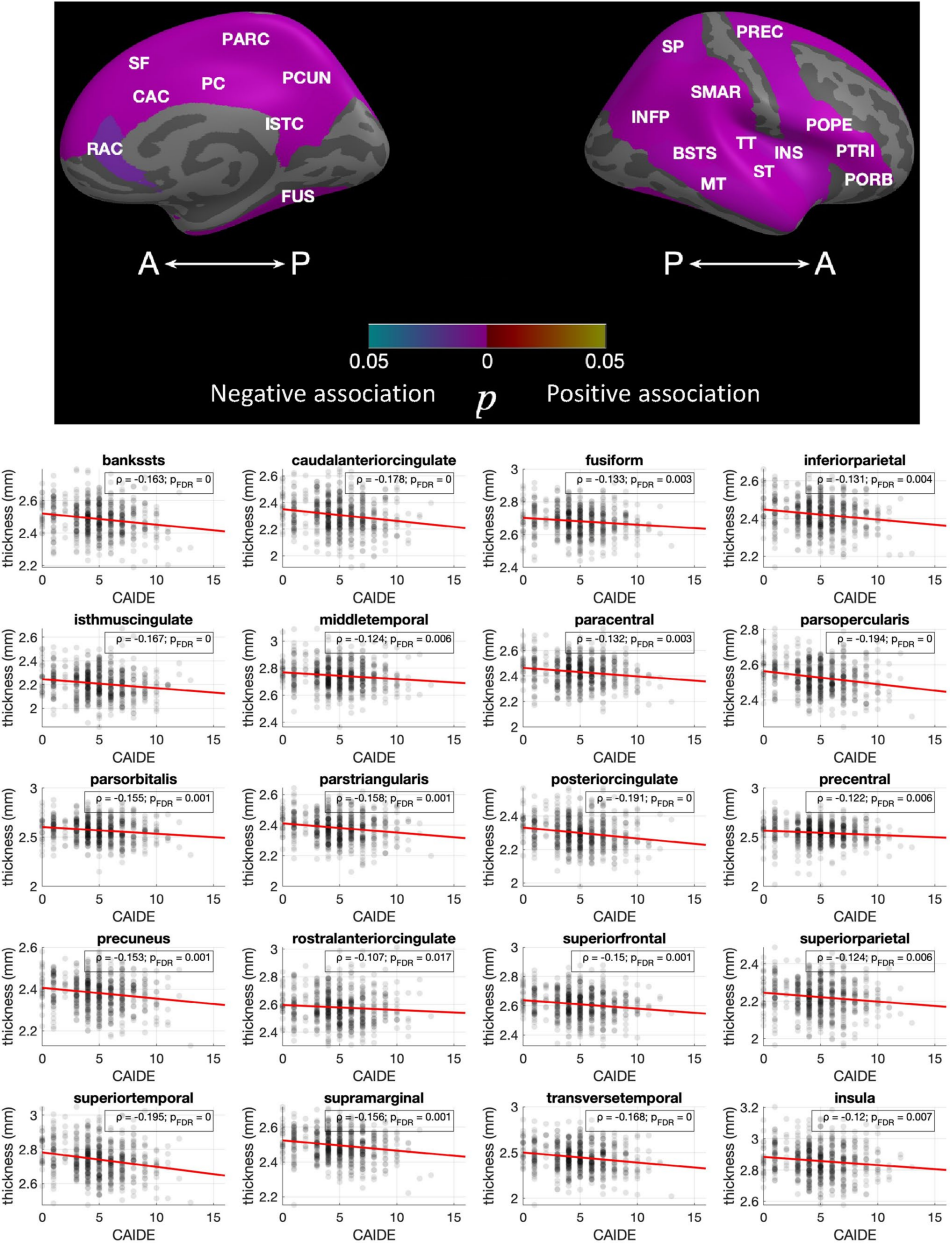
Associations of CAIDE with regional thickness**.** All the identified associations were negative. The overlayed p-values are based on the conducted Spearman correlations and are FDR corrected at a level of *p* < 0.05.*p*_FDR_ values lower that 0.001 are shown as 0. Scatterplots with cortical thickness on the y axis and CAIDE on the *x* axis are shown with a linear fitting. Overlayed are the *ρ* coefficients along with the FDR corrected *p*-values**.** Abbreviations: BSTS, bank of STS; CAC, caudal anterior cingulate; FUS, fusiform; INFP, inferior parietal; INS, insula; ISTC, isthmus cingulate; MT, middle temporal; PARC, paracentral lobule; PC, posterior cingulate; PCUN, precuneus; POPE, Pars opercularis; PORB: Pars orbitalis; PREC, precentral; PTRI, Pars triangularis; RAC, rostral anterior cingulate; SF, superior frontal; SMAR, supramarginal; SP, superior parietal; ST, superior temporal; TT, transverse temporal. *freesurfer_statsurf_display* was used to visualize statistical results on the brain surface

A higher CAIDE score was associated with a smaller thalamus (*ρ* = − 0.11, *p* = 0.01, *p*_FDR_ = 0.07) and smaller nucleus accumbens (*ρ* = − 0.10, *p* = 0.02, *p*_FDR_ = 0.07), findings which did not remain significant following FDR. Several significant associations were observed between CAIDE and regional cortical thickness. Associations surviving FDR correction at a level of *p* < 0.05 are shown in Fig. 3. There were no differences in the examined brain volumes between APOE4 carriers and non-carriers or between FHD- and FHD+; only the amygdala was slightly larger in FHD+ (*t*_FHD_ = 2.18, *p* = 0.03, *p*_FDR_ = 0.21). Differences in cortical thickness were noticed for two regions between APOE4 carriers and non-carriers and two between FHD− and FHD+ which did not survive FDR, these were: banks of the superior temporal sulcus (*t*_APOE4_ = 2.85; *p* < 0.01, *p*_FDR_ = 0.10), middle temporal gyrus (*t*_APOE4_ = 2.75; *p* = 0.01, *p*_FDR_ = 0.10), post central gyrus (*t*_FHD_ = 2.04, *p* = 0.04, *p*_FDR_ = 0.82) and transverse temporal (*t*_FHD_ = 1.97, *p* = 0.05, *p*_FDR_ = 0.82). In further exploratory analysis based on the number of APOE4 copies, heterozygotes had thicker cortex compared to non-carriers in the banks of the superior temporal sulcus (*t*_APOE4_1_ = 2.96; *p* < 0.01, *p*_FDR_ = 0.11) and in the middle temporal gyrus (*t*_APOE4_1_ = 2.30; *p* = 0.02, *p*_FDR_ = 0.37) and homozygotes only in the middle temporal gyrus (*t*_APOE4_2_ = 2.19; *p* = 0.03, *p*_FDR_ = 0.97).

### Differential effect of risk factors with age

A small number of interactions was observed between the examined risk factors and age, however, none of the observed associations survived multiple comparison corrections, these were as follows: (a) for the volumetric analysis one interaction was observed between FHD and age in the thalamus (*t*_FHD x age_ = 2.01; *p* = 0.05, *p*_FDR_ = 0.32) and (b) for the cortical thickness analysis, the following APOE4 × age associations were observed: precuneus (*t*_APOE4 x age_ = 2.21; *p* = 0.03, *p*_FDR_ = 0.50), superior temporal gyrus (*t*_APOE4 × age_ = 2.18; *p* = 0.03, *p*_FDR_ = 0.50). When examining separately APOE4 homozygotes and heterozygotes, there was only one association for the precuneus (*t*_APOE4_1 × age_ = 2.05; *p* = 0.04, *p*_FDR_ = 0.58).

### Hippocampal subfields

After controlling for eTIV, a higher CAIDE score was associated with a larger hippocampal fissure (*ρ* = 0.14, *p* < 0.01, *p*_FDR_ = 0.01). There were no group differences in the volume of the subfields based on APOE4 or FHD. Further to that, there were no significant interactions of age and risk factors for any of the subfields.

Three raters assessed the clarity of the molecular layer for 10% of the analysable cohort. Cohen’s *κ* was 0.73 for the left hemisphere and 0.79 for the right. The average clarity of the molecular layer was associated with sex (*t*_female_ = 2.23, *p*_female_ = 0.03) and there was also a trend toward a less clear molecular layer for APOE4 carriers (*t*_APOE4_ = − 1.95, *p*_APOE4_ = 0.052), whereas there was no difference based on FHD. When examining clarity in relation to number of APOE4 copies, the effect was similar for homozygotes and heterozygotes (*t*_APOE4_1_ = − 1.63, *p*_APOE4_ = 0.10; *t*_APOE4_2_ = − 1.59, *p*_APOE4_ = 0.11). Interestingly, there was no association between the clarity rating and the volume of the molecular layer. In Spearman correlations between subfield volumes and the molecular layer clarity, clarity was associated with the hippocampal fissure (*ρ* = − 0.14, *p* < 0.01, *p*_FDR_ = 0.01) and hippocampal tail (*ρ* = 0.09, *p* = 0.04, *p*_FDR_ = 0.14). When controlling for eTIV, the average clarity was associated with the CA1 (*ρ* = 0.10, *p* = 0.02, *p*_FDR_ = 0.05), fissure (*ρ* = − 0.15, *p* < 0.01, *p*_FDR_ < 0.01) and hippocampal tail (*ρ* = 0.10, *p* = 0.02, *p*_FDR_ = 0.05) volumes.

## Discussion

In this cohort of middle-aged participants, half with FHD and 39% of whom carry at least one copy of the APOE4 allele, a higher CAIDE score was associated with extensive areas of cortical thinning and a higher hippocampal fissure volume. There were no prominent alterations in the volume of subcortical structures, cortical thickness, hippocampal volume and hippocampal subfield volume associated with APOE4 or FHD. APOE4 carriers had a less clear molecular layer. We have further investigated whether age interacted with APOE4 or FHD in predicting structural measures, however, we found no significant interactions that survived multiple comparison corrections. Additionally, we did not find different patterns of atrophy or thinning based on the number of APOE4 copies. Hence, in this cognitively normal healthy midlife cohort, an estimated 23 years from dementia onset, we found evidence of macrostructural changes only when considering the CAIDE score incorporating lifestyle risk factors.

Structural alterations are considered irreversible and occur late in the AD trajectory. Based on studies on carriers of mutations leading to autosomal dominant forms of the disease, structural alterations in the form of localized atrophy occur approximately five years before the expected disease onset [42]. In the same study, the precuneus showed evidence of cortical thinning 13 years before the expected onset. Conversely, in presenilin-1 mutation carriers 10 years from the expected onset, cortical thickening has been reported [43]. In this study, we replicated earlier findings from the West London arm of the study of very limited structural alterations when considering APOE4 and FHD [44]. However, a number of significant associations between structural measures (negative for regional cortical thickness and subcortical volumes and positive for the hippocampal fissure) and CAIDE score was observed.

Several of the areas where CAIDE and cortical thickness were negatively associated are areas typically associated with the prodromal AD signature pattern [45]. In PREVENT-Dementia using data from one study site and a binarized CAIDE score (incorporating APOE4), we have found evidence of longitudinal and established atrophy as well as longitudinal ventricular enlargement [46, 47]. Similar findings regarding CAIDE and thickness have previously been reported, in the presence though of hippocampal and GM atrophy [48]. A CAIDE of more than 12 confers a probability of 16.4% for future dementia with scores above 8 associated with a probability of more than 4% [9]. In a study on individuals with a mean age of 46, subjects with a score greater than 8 had a 29% 40-year risk for dementia [10]. Hence, our findings might imply that in participants with a higher CAIDE, AD-like macrostructural patterns are emerging.

In analysis unadjusted for multiple comparisons, we found that APOE4 carriers appear to have regionally thicker cortex; positive interactions were also observed between APOE4 carriership and age in predicting regional thickness. Even though the observed associations did not remain significant following multiple comparisons correction, and so should be interpreted with caution, they are indicative of an underlying pattern of a thicker cortex in APOE4 carriers. Thicker cortices in APOE4 young or middle-aged carriers have been observed in the past in cognitively normal populations and in early MCI [22, 49]. In a study by Espeseth et al. it was reported that APOE4 carriers demonstrate higher thickness and an accelerated cortical thinning [21]. Conversely, reduced thickness has been reported in APOE4 carriers in the middle-aged VETSA cohort (mean age 55) [23]. Heterogeneity of findings in the field could be attributed to applied processing approaches as well as on the age range and pathology present in the examined cohorts.

Several large cohorts have investigated volumetric alterations in preclinical AD either using an ROI-based approach or voxel-based morphometry. Subtle structural alterations—both atrophy and hypertrophy—have been found in the ALFA study in APOE4 carriers (mean age 58) [11]. In the AIBL study, there were no group differences between different APOE4 genotypes only a trend toward lower hippocampal volumes in ε4 homozygotes (mean age 70) [50]. When examining independent and joined effects of APOE4 and FHD no differences in GM where reported in a mid-aged population [51].

A positive correlation between CAIDE and hippocampal fissure volume was found. We did not observe any other differences following FDR in the subfields volume with CAIDE or between the compared groups. The fissure is the only subfield increasing in volume in AD [34]. In previous reports of a subsample of the PREVENT-Dementia study we have reported a smaller molecular layer in APOE4 carriers, larger fissure with higher CAIDE [28] and smaller CA1 with CAIDE and in participants with FHD [52]. It needs to be mentioned that a different methodological approach for the automated processing was followed in the present paper (Freesurfer version, quality control, eTIV as an analysis covariate). These findings are in line with studies reporting absence of group-differences based on APOE4 in midlife [16]. In the largest study to date examining hippocampal subfields in 39,695 UK Biobank participants divergence of APOE4 homozygotes from the other groups was prominent after the age of 65 with some subfields diverging after 50 [53]. When examining the clarity of the molecular layer, we found that APOE4 carriers had a poorer molecular layer clarity, potentially a prelude of future atrophy. The molecular layer/SRLM is one of the subfields demonstrating early neurodegenerative patterns according to post-mortem studies and in-vivo imaging [17].

The PREVENT-Dementia study is a longitudinal multi-site study in the UK and Ireland targeted at identifying early biomarkers for Alzheimer’s disease. In this study, we provide early proof that the recruited cohort, with a mean age of 51 years, at the first study time-point, did not demonstrate prominent patterns of structural alteration commonly observed in MCI or AD when genetic risk was considered. It was only when a risk score incorporating lifestyle factors, sex and age was taken into account, that alterations were unraveled. Strengths of this study are its large well-characterized middle-aged cohort which is rarely investigated. State-of-the-art analyses methods and a thorough quality control protocol were applied to the acquired data. Limitations include the cross-sectional nature of the study and the absence of further well-established preclinical biomarkers such as amyloid and tau status.

Overall, in the present study, we have shown that midlife participants with a mean age of 51 years, demonstrate macrostructural alterations with an increasing CAIDE score. When APOE4 genotype or FHD were used for group comparisons, there were no group-differences. Hence, these findings highlight the importance of considering modifiable risk factors when stratifying risk populations or potentially designing randomized control trials. In fact, a randomized multi-domain control trial in the FINGER population (mean age 70 years old) over two years demonstrated that the applied lifestyle and vascular interventions had an impact on cognition, however, brain structure was not influenced potentially due to the fact that macrostructural changes appear to be well-established at this stage [54]. Hence further investigation of pathological alterations in relation to modifiable risk factors in middle-age is warranted to unveil the sequalae of alterations leading to dementia.

## Data Availability

Data are available upon reasonable request.
